# Complete Genome Sequence of the Human Gut Symbiont *Roseburia hominis*

**DOI:** 10.1128/genomeA.01286-15

**Published:** 2015-11-05

**Authors:** Anthony J. Travis, Denise Kelly, Harry J. Flint, Rustam I. Aminov

**Affiliations:** a Rowett Institute of Nutrition and Health, University of Aberdeen, Aberdeen, Scotland, United Kingdom; b Technical University of Denmark, National Veterinary Institute, Frederiksberg, Denmark; c Institute of Biological and Environmental Sciences, University of Aberdeen, Aberdeen, Scotland, United Kingdom

## Abstract

We report here the complete genome sequence of the human gut symbiont *Roseburia hominis* A2-183^T^ (= DSM 16839^T^ = NCIMB 14029^T^), isolated from human feces. The genome is represented by a 3,592,125-bp chromosome with 3,405 coding sequences. A number of potential functions contributing to host-microbe interaction are identified.

## GENOME ANNOUNCEMENT

One of the most proficient butyrate producers in the human gut is *Roseburia hominis* A2-183, which produces up to 20 mM of butyrate (1). In ulcerative colitis (UC) the numbers of *R. hominis* are significantly lower compared to control (2, 3). Its use for nutritional/medical applications has been proposed (D. Kelly, 13 August 2014, WO Patent App. PCT/GB2012/052,495), and it has received an orphan drug designation for UC (http://www.accessdata.fda.gov/scripts/opdlisting/oopd/OOPD_Results_2.cfm?Index_Number=439114). Flagellins from this bacterium are potent immunomodulators (D. Kelly, A. Patterson, E. Monnais, I. Mulder, 16 October 2014, WO Patent App. PCT/GB2014/051,123). We sequenced and annotated its genome to reveal beneficial properties.

The strain was grown as described before (4). Chromosomal DNA was isolated using an UltraClean Microbial DNA isolation kit (MoBio Laboratories) and a Wizard Genomic DNA purification kit (Promega); 1.5- to 3.5-kb fragments were cloned using the CloneSmart LCAmp kit (Lucigen), 4- to 8-kb fragments using the pJAZZ-OC vector (Lucigen), and 40-kb fragments using the CopyControl fosmid library production kit (Epicentre Biotechnologies). End reads were obtained by Sanger sequencing. Genomic DNA was sequenced using 454 GS20/454 FLX sequencers. Reads were assembled using MIRA 3 (http://chevreux.org/projects_mira.html). The RAST (5) and Prokaryotic Genome (6) pipelines were used for annotation and comparative genomics.

The strain harbors a single circular genome of 3,592,125 bp, with a G+C content of 48.5%. We identified 3,285 genes, 3,143 potential protein-encoding sequences, 4 ribosomal operons with 12 rRNA genes, and 57 tRNA genes. The largest gene counts belonged to carbohydrate metabolism (17.6%), biosynthesis of amino acids and derivatives (10.8%), and protein metabolism (10.7%). Comparative genomics identified *Roseburia inulinivorans*, *Roseburia intestinalis*, and *Eubacterium rectale* as closest relatives, which is consistent with their close phylogenetic relationship (7).

*R. hominis* produces butyrate via the acetyl-CoA pathway. The pathway genes are found at four locations: (i) acetyl-CoA acetyltransferase, 3-hydroxybutyryl-CoA dehydrogenase, and butyryl-CoA dehydrogenase, including electron transfer protein α and β subunits; (ii) enoyl-CoA hydratase and butyryl-CoA dehydrogenase, including electron transfer protein α and β subunits; (iii) 3-hydroxybutyryl-CoA dehydratase; and (iv) 4-hydroxybutyrate coenzyme A transferase. The genes for conversion of 3-hydroxy butanoyl-CoA to crotonyl-CoA to butyryl-CoA are present in two nonidentical copies. This contributes to the enhanced butyrate production by eliminating bottlenecks in the conversion of acetyl-CoA to butyrate.

The CRISPR-Cas system in this bacterium includes a 33-bp consensus direct repeat GTCGCTCCCTGTGAGGGGAGCGTGGATTGAAAT with 82 spacers, with a total length of 6,582 bp. The large number of spacers found maybe due to its symbiotic nature: there is a negative correlation between the number of repeats and pathogenic potential (8, 9). CRISPR-Cas acts as a barrier against the gene influx including virulence genes.

Flagellin signaling via TLR5 protects against chemicals, bacteria, viruses, and radiation (10, 11), while its inhibition results in spontaneous colitis (12). Four different flagellin genes present in the genome, but only one resides within the flagellar operon, while the others are located in regions unrelated to flagellar motility. This increased gene dose may contribute to the immunomodulatory and protective properties of this bacterium.

### Nucleotide sequence accession number.

The genome of *R. hominis* A2-183 has been deposited in GenBank under the accession number CP003040.
